# Prevalence and risk factors of thrombotic events on patients with COVID-19: a systematic review and meta‐analysis

**DOI:** 10.1186/s12959-021-00284-9

**Published:** 2021-05-19

**Authors:** Xiaoming Xiong, Jianhua Chi, Qinglei Gao

**Affiliations:** 1 Tongji Hospital, Tongji Medical College, National Medical Center for Major Public Health Events, Huazhong University of Science and Technology, 1095 Jiefang Ave, 430000 Wuhan, People’s Republic of China; 2 Cancer Biology Research Center (Key Laboratory of Chinese Ministry of Education), Tongji Hospital, Tongji Medical College, Huazhong University of Science and Technology, Wuhan, People’s Republic of China; 3 Department of Gynecology and Obstetrics, Tongji Hospital, Tongji Medical College, Huazhong University of Science and Technology, Wuhan, People’s Republic of China

**Keywords:** Thrombosis, COVID-19, Prevalence, Risk factors, Anticoagulants

## Abstract

**Background:**

Coagulation abnormalities in COVID-19 patients accompanied with poor prognosis. This study aimed to determine the prevalence and risk factors of thrombotic events on COVID-19 patients.

**Methods.:**

We systematically reviewed all the studies about thrombotic events on COVID-19 patients in PubMed, Embase, Web of Science, MedRxiv, bioRxiv, from Dec 1, 2019 to July 5, 2020. The weighted mean difference (MD) or odds ratio (OR) or relative risk (RR) with 95 % confidence intervals (CI) for clinical data in COVID-19 patients with or without thrombotic events was calculated.

**Results:**

12 articles contained 1083 patients were included for meta-analysis. The prevalence of thrombosis was 22 % (95 % CI 0.08–0.40) in COVID-19 patients and increased to 43 % (95 % CI 0.29–0.65) after admission to the intensive care unit (ICU). Compared with non-thrombotic patients, thrombotic patients had higher levels of D-dimer (MD = 2.79 μg/ml, 95 % CI 2.27–3.31 μg/ml), lactate dehydrogenase (LDH) (MD = 112.71 U/L, 95 % CI 62.40–163.02 U/L), and white blood cells (WBC) (MD = 1.14 *10^9^/L, 95 % CI 0.47–1.81*10^9^/L) while decreased lymphocytes (MD= -0.20*10^9^/L, 95 % CI -0.38 – -0.02*10^9^/L). Age, platelet counts, and male sex tended to be risks while diabetes tended to be a protection for thrombosis for COVID-19 patients, although no statistical difference was achieved. Finally, patients with thrombosis were at a higher risk of death (OR = 2.39, 95 % CI 1.36–4.20).

**Conclusions:**

Prevalence of thrombosis in COVID-19 patients was high, especially in ICU, though pharmacologic thromboembolism prophylaxis was applied. Therefore, higher levels of D-dimer, LDH, WBC, and decreased lymphocytes needed to be paid close attention to in patients with COVID-19.

## Background

Coronavirus Disease 2019 (COVID-19) is prevalent all over the world since it was first reported in Wuhan, China in December 2019. Up to July 18, there were more than 14 million people infected by severe acute respiratory syndrome coronavirus 2 (SARS-CoV-2) and causing nearly 600,000 deaths around the world. The reproduction number R0 of SARS-CoV-2 was 2.92 [1]. Since most people were with high susceptibility to SARS-CoV-2, the World Health Organization (WHO) announced that COVID-19 was a pandemic [2].

More and more studies revealed patients infected by SARS-CoV-2 were accompanied by abnormal coagulation parameters which predicted poor prognosis [3]. Chen et al [4] found that people who died from COVID-19 had longer activated partial thromboplastin time (APTT), higher-level plasma fibrinogen, and D-dimer compared to survivors. A study about patients with COVID-19 in Wuhan, China, revealed elevated D-dimer was observed in critical patients [5]. A few studies demonstrated decreased platelet count was more common in no-survival patients and patients admitted to the ICU.[4] Liu et al. uncovered that the mortality of patients with thrombocytopenia at diagnosis was three times as high as that of those without thrombocytopenia [6]. The prothrombin time (PT) in severe patients was mild prolonged but APTT of dead patients was significantly longer than that of patients who survived [4]. All these coagulation abnormities were also observed in SARS-CoV-1 and MERS-CoV-1 [7].

The hypercoagulation of patients with COVID-19 might be contributed by inflammatory cytokine storm, platelet activation, endothelial dysfunction, and stasis for a long time [8, 9]. Mostafa et al interpreted that inflammation which occurred a few hours after surgery might be an important contributor to endothelial damage, platelet activation, and the generation of tissue factor-bearing microparticles [10]. Many studies revealed significantly elevated several cytokines in plasma of patients with severe COVID-19 [11] and this phenomenon was mainly contributed to the disordered host immune response to SARS-CoV-2 by involving HIF-1α, and ABL tyrosine kinases pathways [12].

Patients with hypercoagulable state were at high risk of suffering from thrombotic events, including venous thrombosis and arterial thrombosis. Similar to SARS-CoV-1 disease [13], COVID-19 was susceptible to be attacked by thrombotic complications because of a hypercoagulable state. A study reported that alveolar-capillary micro thrombosis was nine times more prevalent in patients who died from COVID-19 than in those who died from influenza [14]. The prevalence rate of thrombotic events for patients with severe COVID-19 ranged from 15.2 to 79 % based on available evidence [15–20]. However, there were few studies systematically analyzing the prevalence rate and the risk factors of thrombotic complications for patients with COVID-19. Here we summarized the prevalence and risk factors of thrombotic events for patients with COVID-19 of different countries.

## Methods

### Search strategies and inclusion criteria

We conducted this meta-analysis based on the ‘Preferred Reporting Items for Systematic Reviews and Meta-Analyses (PRISMA)’ statement [21]. We searched for the studies about thrombotic events on patients with COVID-19 in PubMed, Embase, Web of Science, MedRxiv, and bioRxiv, from Dec 1, 2019 to July 5, 2020, using the medical subject heading (MeSH) terms of ‘2019-nCoV’, ‘NCIP’, ‘COVID-19’ or ‘SARS-CoV-2’ and ‘thrombosis’. We furthermore reviewed the references of selected studies in case of omission.

Studies included in this meta-analysis should meet the following criteria: (1) patients were confirmed SARS-CoV-2 infection. (2) only original case series, cohort studies, and retrospective studies reported thrombotic events on patients with COVID-19 could be included. (3) studies must contain clinical characteristics or laboratory tests of patients. (4) the population of studies should be more than 10 cases.

### Data collection

Titles were first screened by two authors (XXM and CJH) separately. If we could not decide to involve potential studies or not according to titles, we further reviewed the abstracts or full-text to make the final decision. The items needed to collect from the selected studies were the first author’s name, the countries of selected studies, mean age of the patients, sex, clinical characteristics, laboratory tests, and outcomes in patients with and without thrombotic complications. Continuous variables presented by median and the interquartile range (IQR) were converted into mean and standard deviation (SD) with the online tool Mean-Variance Estimation (http://www.math.hkbu.edu.hk/~tongt/papers/median2mean.html)[22, 23]. Conflicts about studies selection and data collection were solved by a discussion with another author. Bin Ren et al [15] divided patients with COVID-19 into three groups: No deep venous thrombosis (DVT), isolated distal DVT, and proximal DVT. Therefore, we could not synthesize the laboratory tests from Isolated distal DVT and proximal DVT. Simon et al., [24] a case-control study, recorded the lab-test results by median and IQR but without the first and third quartile, thus we could not transform the data into mean and SD. Thus, we excluded these two studies when we conducted a continuous variable analysis.

### Quality assessment

To assess the quality of included studies, two authors reviewed each study independently and scored them according to the Strengthening the Reporting of Observational Studies in Epidemiology (STROBE) [25].

### Statistical analysis

In this meta-analysis, we calculated the pooled prevalence of thrombotic complications in critical patients with COVID-19 and patients without classification with R software (Version 3.6.2). The prevalence of thrombotic complications in the case-control study was 21/274 [24]. We conducted the analysis of risk factors for thrombotic complications with Review Manager 5.3 (Cochrane IMS, Copenhagen, Denmark). Effect sizes were presented by MD for continuous variables and OR or RR for dichotomous variables with 95 % CI. Since not all included studies reported all variables, we carried out the analysis only with available data for each variable. The results were displayed using forest plots. The heterogeneity between studies was quantified by I^2^ index. If I^2^ < 50 %, a Mantel-Haenszel fixed-effects model was applied. Otherwise, a random-effects model was used.

## Results

### Study selection and characteristics

**Fig. 1:**
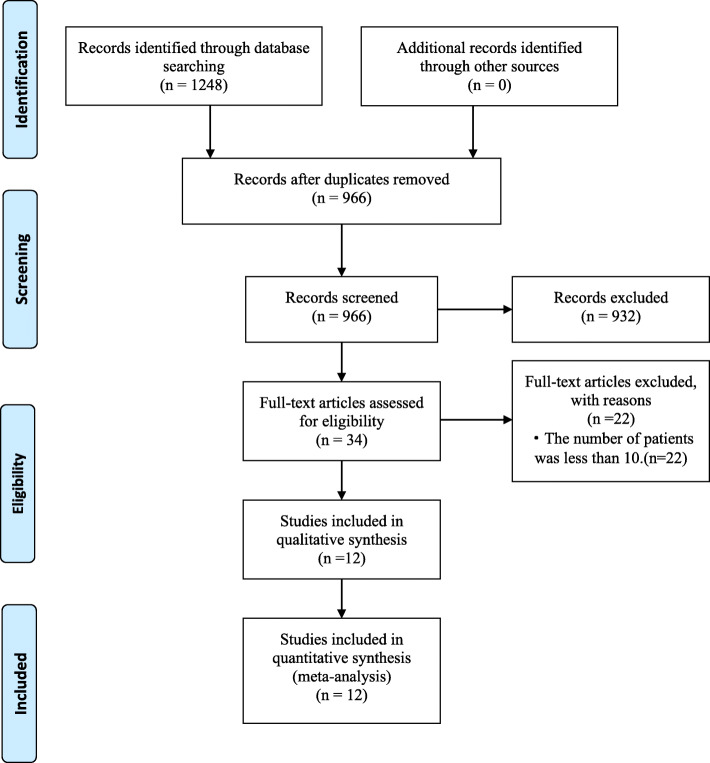
PRISMA flowchart of included studies

Figure 1 shows the study selection process. We initially retrieved 1248 articles using the search strategy, leaving 966 articles after removing duplication. Screening by titles and abstracts, 34 articles were selected full-text assessment. 22 articles were excluded because the population of patients infected with SARS-CoV-2 in these articles was less than 10. Meeting the inclusion criterias, 12 articles and 1083 patients were included for quantitative meta-analysis [15–20, 24, 26–30]. The main characteristics of the included studies were shown in Table 1. These studies enrolled patients from France, China, England, and other countries, with small sample sizes ranging from 34 to 198. Among these included studies, eight were retrospective study, three were prospective study, and left one was case series and case-control study.

**Table 1:** Characteristics of the included studies

Study	Year	Research type	Country	Number of patients		Age median, y		Female n, (%)		Quality Analysis Score
				**Thrombotic**	**Non-thrombotic**	**Thrombotic**	**Non-thrombotic**	**Thrombotic**	**Non-thrombotic**	
**Li Zhang **[26]	2020	Retrospective study	China	66	77	67	59	30 (45.5 %)	39 (50.6 %)	22
**Bin Ren **[15]	2020	Retrospective study	China	41	7	NA	NA	17 (41.5 %)	5 (71.4 %)	20
**Sebastian Voicu **[16]	2020	Prospective study	France	26	30	NA	NA	NA	NA	20
**Michael J R Desborough **[17]	2020	Retrospective study	England	10	56	54	59.3	2 (20 %)	16 (28.6 %)	21
**P. Demelo Rodríguez **[27]	2020	Prospective study	Spain	23	133	66.7	68.4	9 (39.1 %)	35 (26.3 %)	21
**Saskia Middeldorp **[28]	2020	Retrospective study	The Netherlands	39	159	62	60	12 (30.8 %)	56 (35.2 %)	22
**Songping Cui **[18]	2020	Retrospective study	China	20	61	68.4	57.1	NA	NA	20
**Mathieu Artifoni **[29]	2020	Retrospective study	France	16	55	60.2	62.1	5 (31.3 %)	23 (41.8 %)	22
**Megan Fraissé **[19]	2020	Retrospective study	France	37	55	62.4	61.7	8 (21.6 %)	11 (20 %)	21
**Simon M Stoneham **[24]	2020	Case series and case-control study	England	21	42	67	65	7 (33.3 %)	18 (42.9 %)	21
**Julien Nahum **[20]	2020	Prospective study	France	27	7	62.9	59.9	7 (25.9 %)	2 (22.2 %)	21
**Oleg B **[30]	2020	Retrospective study	Russia	15	50	62	69.1	NA	NA	19

*NA* Not available

There was a certain degree of heterogeneity among these studies. Six studies detailed patients who needed mechanical ventilation or were in ICU or seriously ill, one studies reported patients from the general ward, and the other five studies included patients both in the general ward and intensive care units. Almost all patients were treated with thromboembolism prophylaxis using low molecular weight heparin or nadroparin. These studies mainly focus on venous thromboembolism, especially deep venous thrombosis, while only one article reported both venous thrombosis and arterial thrombosis [19].

### Quality Assessment

The total score of quality assessment of included studies was shown in Table 1 according to the STROBE guideline [25]. The overall score of the included studies ranged from 19 to 22, which indicated the high quality of these studies.

### Prevalence of thrombosis in patients with COVID-19

**Fig. 2:**
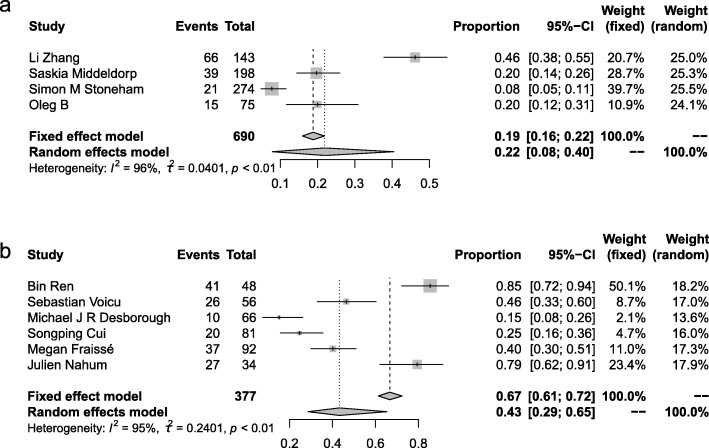
Prevalence of thrombosis in patients with COVID-19. **a**, Overall prevalence of thrombosis in hospitalized patients with COVID-19. **b**, Prevalence of thrombosis in patients with COVID-19 in ICU

As mentioned above, the conditions of patients were not completely consistent in different studies, and admission to the ICU was related to an increased risk of thrombosis. Therefore, we calculated the prevalence of thrombosis twice (Fig. 2). The prevalence is 22 % (95 % CI 0.08–0.40) in patients infected with SARS-CoV-2, and increased to 43 % (95 % CI 0.29–0.65) after admission to ICU, both by the random-effects model.

### Risk factors associated with thrombosis for patients with COVID-19

**Fig. 3:**
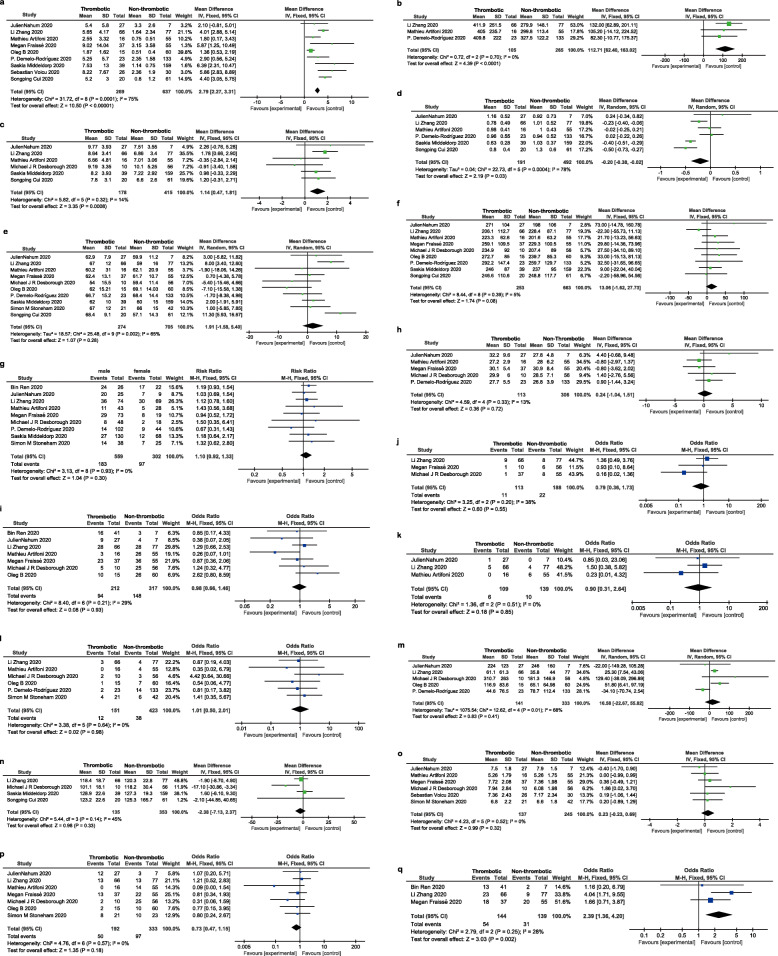
Risk factors analysis of thrombotic events for COVID-19 patients Thrombotic risk of D-dimer (**a**), LDH (**b**), WBC (**c**), lymphocytes (**d**), age (**e**), platelets count (**f**), gender (**g**), BMI (**h**), hypertension (**i**), coronary artery disease (**j**), current-smoking (**k**), malignancy (**l**), CRP (**m**), hemoglobin (**n**), fibrinogen (**o**), and diabetes (**p**) for COVID-19 patients. **q**, The risk of motality for COVID-19 patients with thrombotic events. Note: Heterogeneity is defined according to the I^2^ index calculated. Random or fixed effect models were used base on the heterogeneity

Meta-analysis was carried out on several factors related to thrombosis, and the results are shown in Fig. 3. Compared with non-thrombotic patients, thrombotic patients had higher level of D-dimer (MD = 2.79 μg/ml, 95 % CI 2.27–3.31μg/ml), LDH (MD = 112.71 U/L, 95 % CI 62.40–163.02 U/L), and WBC (MD = 1.14*10^9^/L, 95 % CI 0.47–1.81*10^9^/L) while decreased lymphocytes (MD= -0.20*10^9^/L, 95 % CI -0.38 – -0.02*10^9^/L) (Fig. 3a - d). Age (MD = 1.91 years, 95 % CI -1.58–5.40 years), platelets count (MD = 13.06 *10^9^/L, 95 % CI -1.62–27.73 *10^9^/L), and male (RR = 1.10,95 % CI 0.92–1.33) tended to be risk factors of thrombotic complications for COVID-19 patients though P > 0.05 (Fig. 3e -g). Increased thrombotic complications were not observed in patients with obesity (body mass index (BMI) MD = 0.24, 95 % CI -0.14–1.51), hypertension (OR = 0.98, 95 % CI 0.66–1.46), coronary artery disease (OR = 0.79, 95 % CI 0.36–1.73), current-smoking (OR = 0.90, 95 % CI 0.31–2.64) and malignancy (OR = 1.01, 95 % CI 0.50–2.01) (Fig. 3 h-l). C-reactive protein (CRP) (MD = 16.58 mg/L, 95 % CI -22.67–55.82 mg/L), hemoglobin (mean difference=-2.38 g/L, 95 % CI -7.13–2.37 g/L), and fibrinogen (MD = 0.23 g/L, 95 % CI -0.23–0.69 g/L) did not show significant difference between thrombotic and non-thrombotic patients with COVID-19 (Fig. 3 m-o). Diabetes (OR = 0.73, 95 % CI 0.47–1.15) tended to be protective factors in our study (Fig. 3p). Finally, consisted with our knowledge, patients accompanied by thrombotic events were at higher risk of death (OR = 2.39, 95 % CI 1.36–4.20) (Fig. 3q).

## Discussion

COVID-19 was raging all over the world for the fact that more than 14 million people were infected by SARS-Cov-2 and nearly 600,000 patients died from the disaster till July 18. Therefore, the WHO declared that COVID-19 was a pandemic [2]. COVID-19 was more likely to a multi-system disease which could cause cardiac dysfunction, [31] liver dysfunction, [32] acute kidney injury (AKI), [33] and coagulation disorder [3]. However, the prevalence of thrombotic complications was not systematically well characterized. To the best of our knowledge, this is the first systematic review and meta-analysis on the prevalence and risk factors of thrombosis in patients with COVID-19. Our results show the high prevalence of thrombotic complications in patients with COVID-19, especially in patients with severe conditions admitted to ICU, even in the cases of widespread use of anticoagulation prevention. However, the number of studies on thrombotic events in patients with COVID-19 was limited, and the results varied from study to study. This heterogeneity was not only due to the differences in the aforementioned study population and outcome events, but also due to the fact that thrombus screening in most studies was conducted in patients with venous thrombosis-related symptoms. Wichmann et al [34] discovered that 7 of 12 patients (58 %) were confirmed venous thrombosis by autopsy. What’s more, none of the patients who were diagnosed with venous thrombosis after death was accompanied by thrombosis-related symptoms. A study in Europe revealed the microthrombus in the lungs of patients who died from COVID-19 was ten times as much as those in the lungs of patients who died from influenza (H1N1) [14]. All evidence above suggested that the incidence of thrombotic complications in patients with COVID-19, especially in critical cases, was much higher than that reported in available researches.

Meta-analysis showed that patients with thrombotic complications had higher D-dimer, LDH, WBC, but lower lymphocyte levels than non-thrombosis patients after SARS-Cov-2 infection. This supported the necessary routine monitoring of D-dimer, LDH, WBC, and lymphocytes in thrombosis management of patients with COVID-19. The changes of D-dimer, LDH, and WBC were also closely related to the severity of the patients’ condition and poor prognosis, [35–37] which showed the consistency between thrombosis and adverse outcomes, as higher mortality was observed in patients with thrombosis than those without thrombosis in this study. Some articles had demonstrated evaluated LDH was a predictor for pump-induced thrombosis in patients with continuous-flow left ventricular assist devices [38]. However, it was the first time to identified LDH as a risk factor for thrombosis in patients with COVID-19 in our study. Besides, we also analyzed the relationship between BMI, malignancy, and thrombosis, respectively. To our surprise, obesity and malignancy, which were established risk factors for thrombosis, [39, 40] were not verified in our study. The role of obesity and malignancy in thrombosis for patients with COVID-19 needed to be confirmed by further large-scale study. Though there was no significant relationship between hypertension, as well as diabetes mellitus, and thrombosis in patients with COVID-19, they tended to be protective factors in our study. It might be partially due to the drugs they took such as statins and metformin which had anti-inflammatory effects [41, 42]. We also noted that elder patients were more susceptible to thrombosis and males were at higher risk of developing thrombosis than females, even though the P-value was not significant. A previous study showed aging gave rise to an evaluated incidence of venous thromboembolism (VTE) because of the high level of reactive oxygen species which could injure vascular endothelial cells [43]. Higher COVID-19 mortality in males was reported in 37 of the 38 countries, which showed the average male case fatality rate was 1.7 times higher than the average female case fatality rate [44]. It supported our data that males with COVID-19 were at higher risk of developing thrombosis which might result in higher mortality, consistent with the fact that the patients with thrombotic events were at higher risk of death compared with those without thrombotic events.

Because of the high prevalence rate of thrombosis which resulted in a poor prognosis for patients with COVID-19, it urged us to assess the risk of thrombosis in patients with SARS-Cov-2 infection. Zhai et al[45] recommended all patients needed to undergo dynamic and repeated risk assessment for VTE and bleeding with laboratory monitoring, concomitant medications, invasive procedures, and previous medical history, especially for severe and critically ill COVID-19 patients. Pharmacologic VTE prophylaxis for all hospitalized patients who were confirmed or highly suspected COVID-19 was recommended unless there were contraindications [46]. In accordance with recommendations, amost all patients enrolled in this meta-analysis received anticoagulant prophylaxis with different anticoagulants in hospital. The dose of anticoagulants was adjusted by renal failure and body weight. But the duration of anticoagulant prophylaxis or anticoagulant therapy was not reported in the evaluated studies. The first line prevention for patients with low risk of bleeding was low molecular weight heparin (LMWH) or unfractionated heparin (UFH) for patients with renal dysfunction [45] A lower 28-day mortality was observed in the COVID-19 patients receiving heparin treatment for 7 days or longer according to Tang and his colleagues’ research [47]. Paranjpe et al [48] also reported the in-hospital mortality was 29.1 and 62.7 % for mechanically ventilated patients who were treated with and without anticoagulation separately. All evidence above demonstrated pharmacologic VTE prophylaxis could improve the prognosis of patients with COVID-19, especially critical cases, through inhibiting thrombogenesis or other mechanisms needed further confirmation. And our results provided key items for thrombus monitoring. However, the risk of bleeding was also increased by pharmacologic thrombosis prevention, and the optimal dosing of therapeutic anticoagulation in patients with severe COVID-19 warranted further prospective investigations. It might be useful to start preventive anticoagulation at the early stage of the disease since it was reported that a large number of thromboembolic events were already broken within 24 h of admission [49]. Furthermore, some other drugs such as statins that had a strong effect on anti-inflammation and endothelial protection might take effect in thrombosis prevention base on the evidence that endothelial dysfunction and inflammation initialized thrombosis [50].

The studies involved in this meta-analysis were limited. The thrombosis events and population in different studies were not the same. Though we converted median and IQR into mean and SD by online web tool, minor inaccuracy certainly existed. Besides, mainly included studies were retrospective and coagulation-related biomarkers were not fully reported. Given the limitations above, the results of the meta-analysis should be interpreted carefully.

## Conclusions

The prevalence rate of thrombosis was higher in patients with COVID-19 than we thought, especially in those admitted to ICU, though pharmacologic thromboembolism prophylaxis was applied. Compared with non-thrombotic patients, thrombotic patients had a higher level of D-dimer, LDH, and WBC while decreased lymphocytes. Age, platelets count, and male sex tended to be risk factors of thrombotic complications for COVID-19 patients, notwithstanding statistic difference was not significant in our study. However, diabetes mellitus was inclined to be a protective factor of thrombotic complication for COVID-19 patients in our study, though P > 0.05, which needed to be confirmed by further larger scale of studies. We finally found patients with thrombotic events were at higher risk of death compared with non-thrombotic patients. It was necessary to assess the risk of thrombosis and bleeding for the balance of pharmacologic thromboembolism prophylaxis to improve the prognosis of patients with COVID-19, especially for severe cases. All results provided some risk factors that needed to be paid close attention to for thrombus monitoring in patients with COVID-19.

## Data Availability

All data used for the systematic review and meta-analysis is present in the main manuscript in Table 1; Figures 1-3.

## Abbreviations

APTT: Activated partial thromboplastin time
BMI: Body mass index
CI: Confidence intervals
COVID-19: Coronavirus Disease 2019
CRP: C-reactive protein
ICU: Intensive care unit
IQR: Interquartile range
LDH: Lactate dehydrogenase
LMWH: Low molecular weight heparin
MD: Mean difference
MeSH: Medical subject heading
OR: Odds ratio
PRISMA: Preferred Reporting Items for Systematic Reviews and Meta-Analyses
RR: Relative risk
SARS-CoV-2: Severe acute respiratory syndrome coronavirus 2
SD: Standard deviation
STROBE: Strengthening the Reporting of Observational Studies in Epidemiology
UFH: Unfractionated heparin
VTE: Venous thromboembolism
WBC: White blood cells
WHO: World Health Organization
